# Problematic Pornography Use Among Swedish Adolescents: Characteristics and Clinical Implications

**DOI:** 10.1007/s10508-026-03544-9

**Published:** 2026-10-08

**Authors:** Marta Piwowar, Linda S. Jonsson, Marie Bladh, Åsa Landberg, Carl Göran Svedin, Josephine Savard

**Affiliations:** 1 Department of Medicine, Huddinge, Karolinska Institutet, Stockholm, Sweden; 2 Department of Social Sciences, Marie Cederschiöld University, Stockholm, Sweden; 3 Department of Obstetrics and Gynecology in Linköping and Division of Children’s and Women’s Health, Department of Biomedical and Clinical Sciences, Faculty of Medicine and Health Sciences, Linköping University, Linköping, Sweden; 4 Department of Clinical Sciences (Psychiatry), Umeå University, Umeå, Sweden; 5 ANOVA, Norra Stationsgatan 69, 113 64 Stockholm, Sweden

**Keywords:** Problematic pornography use, Adolescents, Compulsive sexual behavior disorder, Mental health, Sexual behaviors

## Abstract

Problematic pornography use (PPU) is the most common manifestation of compulsive sexual behavior disorder (CSBD). Many adults seeking help for CSBD report onset during adolescence, yet there is limited knowledge of PPU in adolescents and its predisposing factors. This study investigated the prevalence of self-perceived PPU among Swedish adolescents and explored associated factors as well as pornography use patterns. In this cross-sectional study, data were collected from a representative sample of 3106 Swedish third-year senior high school students. Information on sociodemographic background, pornography use, sexual behaviors, health, and experiences of abuse was collected. PPU was based on self-reported difficulties in refraining from or stopping pornography use. Gender-stratified descriptive statistics and logistic regression analyses were used to examine relationships between groups and variables. PPU was reported by 10.4% of respondents, with a higher prevalence among boys (18.8%) compared to girls (3.9%). It was associated with increased time spent on pornography and higher exposure to violent or other concerning sexual content. Compared to the reference group, adolescents with PPU exhibited higher rates of abusive experiences, mental health issues, risky behaviors, and nonsuicidal self-injury. Gender-specific patterns emerged. PPU is a significant issue among Swedish adolescents, particularly boys. Addressing concerns related to pornography use, underlying vulnerabilities and gender-specific factors is crucial to prevent the progression of PPU into adulthood and in treatment efforts.

**Supplementary Information:**

The online version contains supplementary material available at https://doi.org/10.1007/s10508-026-03544-9.

## Introduction

Pornography use is common among adolescents, with a higher frequency among males; however, the prevalence varies significantly depending on the cultural context and research methodology employed (Peter & Valkenburg, 2016). Despite concerns regarding the potential negative impacts of pornography (Peter & Valkenburg, 2016), most adolescents do not develop problematic patterns of pornography use (Bőthe et al., 2021; Svedin et al., 2023). Nevertheless, a subset of users faces the risk of developing problematic pornography use (PPU). While there is no universally accepted definition for PPU, it is typically defined as a perceived loss of control over pornography consumption, leading to distress (Bőthe et al., 2018; Efrati, 2020). Impaired control is not only suggested to play a significant role in PPU but can also predict PPU six months later (Chen et al., 2022). A study across 42 countries estimated the prevalence of PPU at 3.2%–16.6% (Bőthe et al., 2024a, 2024b).

Considering that impaired control over pornography use is a central clinical manifestation of compulsive sexual behavior disorder (CSBD, Reid et al., 2012), PPU is regarded as a potential subtype of CSBD (Antons & Brand, 2021; Efrati & Gola, 2018a). CSBD is included in the 11th revision of the *International Classification of Diseases* (ICD-11), defined as a persistent pattern of difficulties in controlling repetitive sexual impulses, urges, and behaviors (Kraus et al., 2018; World Health Organization, 2022). The prevalence of CSBD among youths has scarcely been explored. An online study (Jepsen & Brzank, 2022) found that 10.5% of German young adults aged 18–27 had indications of compulsive sexual behaviors (CSB), with males exhibiting a higher prevalence. The World Health Organization (2022) advises caution in the diagnosis of adolescents, acknowledging that high sexual interest is a normal aspect of sexual development, despite potential distress. Conversely, CSBD can be identified as a distinct condition in young people (Efrati, 2018), rather than a manifestation of other psychopathologies. Additionally, many adults with CSBD report that problematic sexual behaviors began during preadolescence or adolescence (Reid et al., 2012; World Health Organization, 2022). Given the assumption that PPU is associated with CSBD and considering that the onset often occurs during adolescence, it is crucial to identify the problem at an early stage.

Notably, CSBD has been associated with risky sexual behaviors, coercive sexual behaviors, increased exposure to child sexual abuse material, and an elevated risk of sexual re-offending (Bőthe et al., 2020; Engel et al., 2019a, 2019b; Långström & Hanson, 2006). Research suggests that sexual behaviors in CSBD may serve as coping strategies to regulate negative emotions (Lew-Starowicz et al., 2020), which could at least partially explain the common co-occurrence with psychiatric comorbidities (Antons et al., 2023; Turner et al., 2022). Adverse experiences and maltreatment, such as sexual, physical, or emotional abuse, serve as predictors of CSBD (Långström & Hanson, 2006; Slavin et al., 2020) and the risk of CSB significantly increases in the presence of multiple forms of abuse (Kingston et al., 2017). Nonetheless, most studies on predictors of CSBD have been conducted on adults, leaving us with a limited understanding of the predisposing factors and characteristics of adolescents with CSB/PPU.

Among the limited research conducted on youths, studies on Israeli adolescents with CSB revealed more frequent pornography use and online sexual activities (Efrati, 2018, 2023; Efrati & Gola, 2018b). Their profile included higher socioeconomic status, increased religiosity, attachment insecurity, loneliness, an external locus of control, and personality traits such as higher neuroticism. Psychopathological symptoms related to anxiety and a propensity to ruminate were also linked to the severity of CSB. Other research on adolescents and CSB (Doornwaard et al., 2016; Štulhofer et al., 2020) demonstrated a negative correlation with life satisfaction and psychological well-being. Additionally, male gender, problematic pornography consumption, and impulsivity emerged as distinct predictors of CSB in young adults (Jepsen & Brzank, 2022).

In Sweden, cultural norms regarding sexuality are often characterized by comparatively liberal attitudes; sexual education is designed to be inclusive and norm-aware, encouraging critical reflection on sexuality, consent, relationships, and the influence of pornography (Skolverket, 2024). Over the past two decades, while overall engagement with pornography among Swedish adolescents has decreased, the proportion of boys consuming it weekly has risen from 11 to 24% (Donevan et al., 2022). However, the designation of PPU should not be based solely on the frequency of sexual behavior (Bőthe et al., 2018), but rather on the experience of loss of control. Therefore, this study focuses on adolescents’ self-perceived difficulties in stopping or refraining from pornography use. While previous studies have primarily examined correlates of PPU in adults, little is known about the characteristics and the predictive strength of a broad spectrum of correlates within a nationally representative sample of adolescents in a secular context. Identifying factors associated with PPU could have clinical implications for the early recognition of youths with PPU and the prevention of potential progression into CSBD in adulthood.

### Aim

One aim of this study is to investigate the prevalence of self-perceived PPU among Swedish adolescents and to explore possible correlates of PPU, such as sociodemographic factors, sexual experiences, health, and adverse experiences. Another aim is to investigate how pornography use differs between adolescents with and without an indication of PPU.

## Method

### Participants and Procedure

The data collection was part of the fourth national study on adolescent sexuality and exposure to sexual assault and exploitation (Svedin et al., 2021). A representative and stratified sample of 210 schools was selected based on study program and school size, using the high school register of the Swedish National Agency for Education. Data collection took place between December 2019 and March 2021, with a break due to the COVID-19 pandemic. The study transitioned from classroom-based data collection to a digital solution enabling remote participation during the pandemic.

**Fig. 1:**
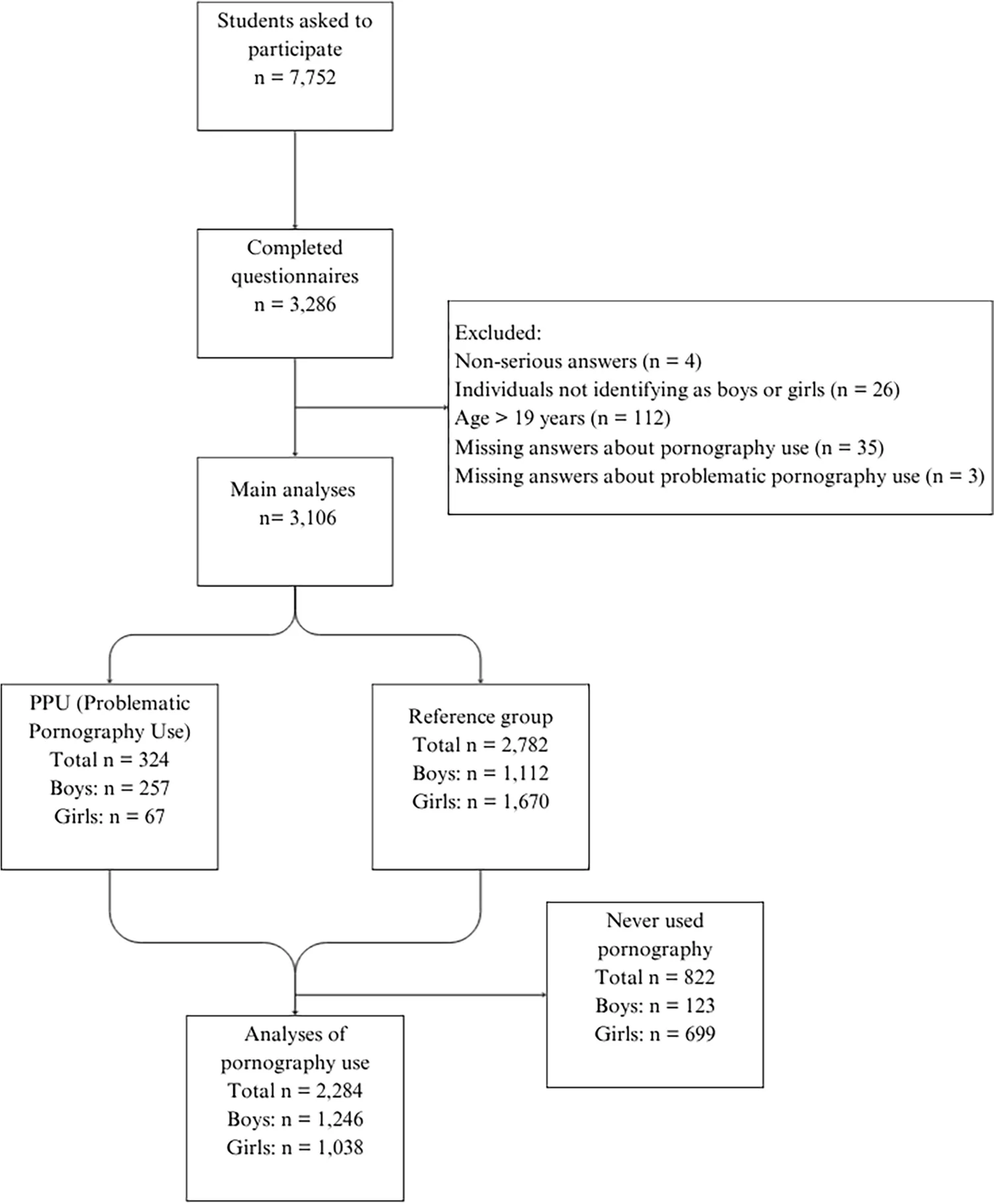
Participant flow diagram

This study included students: (1) who answered all questions on pornography use with coherent responses; (2) who were aged ≤ 19 years; and (3) who identified as either male or female, with non-binary individuals being too few for statistical analyses. The final sample consisted of 3106 participants, 1,369 boys (44.1%) and 1,737 girls (55.9%), with a median age of 18 (range 16–19) (Fig. 1).

### Measures

The survey included 112 main questions, encompassing questions on sociodemographic background, disability/chronic illness, sexuality, alcohol and substance use, abusive experiences, psychological well-being, and internet and pornography use. In this study we included variables likely to impact PPU as presented in the introduction.

Pornography was defined as pictures or films where one or more people have sex with themselves or with each other. Individuals who reported ever watching pornography answered additional questions on their pornography use. Participants who found it “difficult to refrain from watching pornography,” “difficult to stop watching pornography once I started,” or both (i.e., ≥ 4 on a 5-point Likert scale from 1 = *totally disagree* to 5 = *totally agree*) were classified as having PPU. Participants rated their pornography use frequency over the past year. The two lowest-frequency options were combined to “up to some time each month,” while the remaining options were “some time each week” and “more or less daily.” Similarly, for the variable “time spent on pornography use/time,” the two highest time options were merged into “more than 1 h,” with the remaining options being “30–59 min” and “less than 30 min.” Participants reported any experience with violent/coercive pornography and with sexual abuse material involving children under 15 or animals (presented here as a merged variable, “ever used sexual material involving children or animals”).

#### Sociodemographic Factors

Sociodemographic factors included living situation (living with both parents/alternating or not), place of birth (born in Sweden or not), parents’ employment (employed mother or not, employed father or not), perceived family economy (poor economic situation or not), and study program (theoretical, vocational, or individual).

#### Sexuality

Sexual identity was initially categorized by five answer options, and later dichotomized into heterosexual and other. Experience of oral, vaginal, and anal sex was reported irrespective of partner gender. A binary variable “exposed someone else to nonconsensual sexual acts” summarized various nonconsensual offline sexual behaviors. The age at first voluntary sexual experience was dichotomized into < 15 years/≥ 15 years, in line with Sweden’s legal age of consent. The number of sexual partners (categorized into 0, 1–5, and ≥ 6), history of chlamydia, abortion, and experience of sending sexually explicit images (all dichotomized into no/yes) were also included.

#### Health and Abusive Experiences

High-risk alcohol consumption was defined according to former national guidelines (Socialstyrelsen, 2018) as ingesting 4 (women) or 5 (men) glasses (i.e., units, where 1 unit = 12 g alcohol) on the same occasion at least once a month. Drug use was dichotomized into ever used drugs versus never used drugs. The variable “Psychiatric/neuropsychiatric comorbidity” was constructed based on participants’ self-reported diagnoses made by a medical doctor or psychologist. Included conditions were substance use disorder, eating disorder, depression/anxiety, ADHD/ADD, autism, and Tourette’s disorder. Single-item questions assessed the lifetime prevalence of nonsuicidal self-injury and the use of sex as self-injury.

Any lifetime bullying experience was coded as “exposure to bullying.” The variables of emotional and physical abuse included any exposure to such abuse by an adult before the age of 18 years. Sexual abuse was defined by the question “People can be pressured, convinced, or forced to participate in sexual acts that they cannot defend themselves against. Have you ever been exposed to any of the following?” followed by examples of sexual abuse ranging from non-contact behaviors to penetrative abuse. “Multiple types of abuse” indicated that a participant was exposed to more than one type of abuse, including bullying. Exposure to grooming was based on examples of internet grooming behaviors presented in the survey, prior to the age of 15, by an individual at least 5 years older. All variables were dichotomized into yes or no.

#### Questionnaires

Difficulties with the* Emotion Regulation Scale (DERS-16)* (Bjureberg et al., 2016). The scale consists of 16 items rated on a 5-point Likert scale, ranging from *almost never* to *almost always*. Scores range from 16–80, where a higher score indicates greater emotion regulation difficulties. The scale has demonstrated a Cronbach’s alpha of 0.92.

Trauma Symptom Checklist for Children (TSCC) (Briere, 1996) assesses trauma-related symptoms in children and adolescents, with higher scores indicating worse mental health. Responses are rated on a scale ranging from *never* to *almost always*, with a total scale score range from 0 to 162. A Swedish validation resulted in a Cronbach’s alpha of 0.94 (Nilsson et al., 2008) and confirmed TSCC’s effectiveness in distinguishing trauma symptoms in children with traumatic experiences.

### Statistical Analysis

The data were analyzed using descriptive statistics, presented as frequencies, percentages, median values, and interquartile range (IQR). To compare group differences, non-parametric tests were applied: Pearson’s Chi-square test and Fisher’s Exact Test for ordinal data, and the Mann–Whitney *U* Test for continuous variables. Effect sizes were quantified using Phi, rank-biserial correlation coefficient, and Cramer’s V. Univariate and multivariable logistic regression analyses were performed to identify the factors associated with the dependent variable (PPU). The variables were categorized into three domain-based models: sociodemographic factors, sexuality, and health and abusive experiences. Each domain was initially analyzed separately, omitting variables associated with pornography use due to the distinct reference group. Variables that reached statistical significance in either univariate or multivariate analyses in each domain were included in a final full model. Analyses were stratified by gender. Statistical analyses were performed using the SPSS version 29.0 (IBM Corp., Armonk, NY, USA). All *p*-values were two-tailed, and values < 0.05 were considered statistically significant.

## Results

PPU was reported by 324 participants (10.4%): 257 boys (18.8%) and 67 girls (3.9%) (Fig. 1).

### Sociodemographic Factors

No significant differences were observed in sociodemographic factors between PPU and reference groups (Table 1).

**Table 1:** Sociodemographic factors: Comparisons between Problematic Pornography Use (PPU) group (*n* = 324) and Reference group (*n* = 2782)

	Boys		Girls			
	PPU	Reference group	PPU	Reference group	Boys PPU vs. Reference group	Girls PPU vs. Reference group
	*n* = 257	*n* = 1,112	*n* = 67	*n* = 1,670	*n* = 1,369	*n* = 1,737
	*n* (%)	*n* (%)	*n* (%)	*n* (%)	Statistics *χ*^2^, *p, φ*	Statistics *χ*^2^, *p, φ*
Living with both parents or alternating	194 (75.5)	853 (76.7)	50 (74.6)	1,230 (73.7)	*χ*^2^ = .11, *p* = .738	*χ*^2^ = .00, *p* = .971
					*φ* = .01	*φ* = .00
Born in Sweden	228 (88.7)	1,016 (91.4)	56 (83.6)	1,524 (91.3)	*χ*^2^ = 1.46, *p* = .226	*χ*^2^ = 3.73, *p* = .053
					*φ* = − .04	*φ* = − .05
Employed mother	230 (89.5)	988 (88.8)	58 (86.6)	1,467 (87.8)	*χ*^2^ = .04, *p* = .852	*χ*^2^ = .02, *p* = .902
					*φ* = .01	*φ* = − .01
Employed father	224 (87.2)	996 (89.6)	54 (80.6)	1,446 (86.6)	*χ*^2^ = 1.01, *p* = .314	*χ*^2^ = 1.49, *p* = .223
					*φ* = − .03	*φ* = − .03
Poor economic situation	12 (4.7)	33 (3.0)	4 (6.0)	59 (3.5)	*χ*^2^ = 1.40, *p* = .236	FET: *p* = .303
					*φ* = .04	
*Study program*						
Theoretical	201 (78.2)	820 (73.7)	49 (73.1)	1,201 (71.9)	*χ*^2^ (2) = 4.33, *p* = .115	FET: *p* = .936
Vocational	45 (17.5)	257 (23.1)	17 (25.4)	417 (25.0)	Cramer’s* V* = .06	
Individual	11 (4.3)	35 (3.1)	1 (1.5)	52 (3.1)		

Statistics: Pearson’s chi-square statistics (*χ*^2^), Fisher’s Exact Test (FET), Phi (*φ*), Cramer’s *V*

### Sexuality

Table 2 shows the results of sexuality-related variables. Girls with PPU reported having more sexual partners than the reference group (*p* = .009) with 28.4% of girls having had more than six sexual partners, compared to 14.6% in the reference group. Sexual debut < 15 years was also more prevalent among girls with PPU compared to those with no PPU (29.9% vs. 12.9%, *p* < .001).

**Table 2:** Sexuality: Comparisons between Problematic Pornography Use (PPU) group (*n* = 324) and Reference group (*n* = 2782)

	Boys		Girls			
	PPU	Reference group	PPU	Reference group	Boys PPU vs. Reference group	Girls PPU vs. Reference group
	*n* = 257	*n* = 1,112	*n* = 67	*n* = 1,670	*n* = 1,369	*n* = 1,737
	*n* (%)	*n* (%)	*n* (%)	*n* (%)	Statistics *χ*^2^, *p, φ*	Statistics *χ*^2^, *p, φ*
Heterosexual identification	223 (86.8)	1,003 (90.2)	51 (76.1)	1,332 (79.8)	*χ*^2^ = 2.27, *p* = .132	*χ*^2^ = .33, *p* = .568
					*φ* = − .04	*φ* = − .02
*Number of sexual partners*						
0	108 (42.0)	411 (37.0)	18 (26.9)	539 (32.3)	*χ*^2^ = 2.34, *p* = .311 Cramer’s* V* = .04	***χ***^**2**^** = 9.48, *p* = .009** **Cramer’s* V* = .07**
1**–**5	125 (48.6)	582 (52.3)	30 (44.8)	887 (53.1)		
≥ 6	24 (9.3)	119 (10.7)	19 (28.4)	244 (14.6)		
Experience of oral sex	135 (52.5)	619 (55.7)	47 (70.1)	1,031 (61.7)	*χ*^2^ = .71, *p* = .400	*χ*^2^ = 1.60, *p* = .207
					*φ* = − .03	*φ* = .03
Experience of vaginal sex	132 (51.4)	661 (59.4)	46 (68.7)	1,089 (65.2)	***χ***^**2**^** = 5.27, *p* = .022**	*χ*^2^ = .20, *p* = .652
					***φ* = ** − **.06**	*φ* = .01
Experience of anal sex	49 (19.1)	187 (16.8)	19 (28.4)	338 (20.2)	*χ*^2^ = .59, *p* = .442	*χ*^2^ = 2.13, *p* = .145
					*φ* = .02	*φ* = .04
Sexual debut < 15 years	23 (8.9)	122 (11.0)	20 (29.9)	216 (12.9)	*χ*^2^ = .70, *p* = .403	***χ***^**2**^** = 14.29, *p* < .001**
					*φ* = − .03	***φ* = .10**
Sending sexually explicit images	103 (40.1)	297 (26.7)	44 (65.7)	713 (42.7)	***χ***^**2**^** = 17.40, *p* < .001**	***χ***^**2**^** = 12.91, *p* < .001**
					***φ* = .12**	***φ* = .09**
History of chlamydia	3 (1.2)	9 (0.8)	4 (6.0)	41 (2.5)	FET: *p* = .480	FET: *p* = .092
Abortion (self or partner)	3 (1.2)	29 (2.6)	3 (4.5)	31 (1.9)	*χ*^2^ = 1.32, *p* = .251	FET: *p* = .141
					*φ* = − .04	
Exposed someone else to nonconsensual sexual acts^a^	13 (5.1)	12 (1.1)			**FET: *p* < .001**	

Statistics: Pearson’s chi-square statistics (χ^2^), Fisher’s Exact Test (FET), Phi (*φ*), Cramer’s *V*

^a^Only calculated for boys due to low prevalence among girls

Note. Bold values indicate statistically significant results (*p* < .05).

Boys in the PPU group had less experience of vaginal sex compared to those in the reference group (51.4% vs. 59.4%, *p* = .022). More boys with PPU had exposed someone else to offline nonconsensual sexual acts compared to the reference group (5.1% vs. 1.1%, *p* < .001). Regardless of gender, the PPU groups were more likely to send sexually explicit images (*p* < .001), with two-thirds of girls in the PPU group reporting having done so.

### Health and Abusive Experiences

Table 3 shows the results of the associations between health and abusive experiences. High-risk alcohol use was more prevalent among girls with PPU compared to the reference group (44.8% vs. 26.4%, *p* < .001). The prevalence of lifetime drug use was twice as high among girls with PPU compared to those in the reference group (35.8% vs. 17.8%, *p* < .001).

**Table 3:** Health and abusive experiences: Comparisons between Problematic Pornography Use (PPU) group (*n* = 324) and Reference group (*n* = 2782). All values are given as n (%) unless indicated otherwise

	Boys		Girls			
	PPU	Reference group	PPU	Reference group	Boys PPU vs. Reference group	Girls PPU vs. Reference group
	*n* = 257	*n* = 1112	*n* = 67	*n* = 1670	*n* = 1,369	*n* = 1,737
	*n* (%)	*n* (%)	*n* (%)	*n* (%)	Statistics χ^2^, *p, φ*	Statistics χ^2^, *p, φ*
*Health*						
High-risk alcohol consumption	82 (31.9)	370 (33.3)	30 (44.8)	441 (26.4)	*χ*^2^ = .12, *p* = .729	***χ***^**2**^** = 10.09, *p* < .001**
					*φ* = − .01	***φ* = .08**
Lifetime drug use	72 (28.0)	311 (28.0)	24 (35.8)	297 (17.8)	χ^2^ = .70, *p* = .403	***χ***^**2**^** = 12.74, *p* < .001**
					*φ* = − .03	***φ* = .09**
Psychiatric/neuropsychiatric comorbidity	42 (16.3)	139 (12.5)	18 (26.9)	345 (20.7)	*χ*^2^ = 2.36, *p* = .124	*χ*^2^ = 1.15, *p* = .284
					*φ* = .04	*φ* = .03
Nonsuicidal self-injury	55 (21.4)	161 (14.5)	35 (52.2)	594 (35.6)	***χ***^**2**^** = 7.01, *p* = .008**	***χ***^**2**^** = 7.05, *p* = .008**
					***φ* = ** − **.07**	***φ* = ** − **.07**
Using sex as self-injury	6 (2.3)	22 (2.0)	16 (23.9)	135 (8.1)	*χ*^2^ = .01, *p* = .905	***χ***^**2**^** = 18.31, *p* < .001**
					*φ* = − .01	***φ* = ** − **.11**
Difficulties with Emotion Regulation Scale (DERS-16), *median (IQR)*	34 (23**–**48)	27 (20**–**36)	48 (36**–**60)	38 (27**–**52)	***z* = 6.16, *p* < .001,**	***z* = 3.81, *p* < .001,**
					***r* = .17**	***r* = .09**
Trauma Symptom Checklist for Children (TSCC), total score, *median (IQR)*	32 (18**–**45)	20 (10**–**33)	50 (34**–**72)	35 (22**–**50)	***z* = 8.18, *p* < .001,**	***z* = 5.05, *p* < .001,**
					***r* = .22**	***r* = .12**
*Abusive experiences*^*a*^						
Exposure to bullying	165 (64.2)	556 (50.9)	52 (77.6)	1025 (61.4)	***χ***^**2**^** = 14.32, *p* < .001**	***χ***^**2**^** = 6.53, *p* = .011**
					***φ* = .10**	***φ* = .06**
Emotional abuse	55 (21.4)	161 (14.5)	19 (28.4)	321 (19.2)	***χ***^**2**^** = 7.02, *p* = .008**	*χ*^2^ = 2.86, *p* = .091
					***φ* = .07**	*φ* = .04
Physical abuse	63 (24.5)	181 (16.3)	19 (28.4)	278 (16.6)	***χ***^**2**^** = 9.12, *p* = .003**	***χ***^**2**^** = 5.43, *p* = .020**
					***φ* = .08**	***φ* = .06**
Exposed to any form of sexual abuse	34 (13.2)	114 (10.3)	35 (52.2)	584 (35.0)	*χ*^2^ = 1.62, *p* = .203	***χ***^**2**^** = 7.64, *p* = .006**
					*φ* = .04	***φ* = .07**
Multiple types of abuse^b^	81 (31.5)	230 (20.7)	36 (53.7)	618 (37.0)	***χ***^**2**^** = 13.35, *p* < .001**	***χ***^**2**^** = 6.98, *p* = .008**
					***φ* = .10**	***φ* = .07**
Grooming	33 (12.8)	108 (9.7)	34 (50.7)	623 (37.3)	*χ*^2^ = 1.89, *p* = .170	**χ**_**2**_** = 4.39, *p* = .036**
					*φ* = .04	***φ* = .05**

Statistics: Pearson’s chi-square statistics (χ^2^), Fisher’s Exact Test (FET), Phi (*φ*), Mann–Whitney U Test (*z*), rank-biserial correlation coefficient (*r*)

^a^Exposure

^b^Exposure to more than one type of abuse above, including bullying

Note. Bold values indicate statistically significant results (*p* < .05).

Both boys and girls with PPU exhibited more nonsuicidal self-injury compared to the reference groups (both *p* < .05), with a notable difference among girls in the use of sex as self-injury (23.9% vs. 8.1%, *p* < .001). Both PPU groups had higher median total scores concerning emotional regulation difficulties (both *p* < .001) as well as trauma symptoms (both *p* < .001). There were no significant differences concerning psychiatric/neuropsychiatric diagnoses.

Both girls and boys with PPU reported more exposure to bullying, physical abuse, and multiple types of abuse. Boys in the PPU group also reported more emotional abuse compared to boys with no PPU (21.4% vs. 14.5%, *p* = .008). Girls with PPU reported more experience of sexual abuse (52.2% vs. 35.0%, *p* = .006) and were more often exposed to grooming (50.7% vs. 37.3%, *p* = .036) compared to the reference group.

### Pornography Use

All factors related to pornography use differed significantly between PPU and the pornography use reference group, irrespective of gender (Table 4). Nearly 12% of girls with PPU reported daily or near-daily use, in contrast to 1% of girls in the reference group (*p* < .001). Notably, the proportion was much higher among boys, with nearly half in the PPU group reporting more or less daily pornography use, compared to 16.1% in the reference group (*p* < .001). The duration of pornography use also varied significantly, with the PPU groups spending considerably more time on such activities. Differences were also observed in the nature of the pornography consumed. The use of violent/coercive pornography was more prevalent among girls with PPU than those in the reference group (37.3% vs. 11.8%, *p* < .001). A similar pattern was observed among boys (26.1% in the PPU group, 8.7% in the reference group, *p* < .001). The lifetime use of sexual abuse material involving children or animals was significantly higher in both PPU groups, with 10.4% of girls and 8.9% of boys reporting such use, compared to 2.1% of girls and 3.5% of boys in the reference groups.

**Table 4:** Pornography use: comparisons between Problematic Pornography Use (PPU) group (*n* = 324) and pornography use Reference group (no PPU) (*n* = 1,960)

	Boys		Girls			
	PPU	No PPU	PPU	No PPU	Boys PPU vs. No PPU	Girls PPU vs. No PPU
	*n* = 257	*n* = 989	*n* = 67	*n* = 971	*n* = 1,246	*n* = 1,038
	*n* (%)	*n* (%)	*n* (%)	*n* (%)	Statistics χ^2^, *p, φ*	Statistics χ^2^, *p, φ*
*Frequency of pornography use*						
Up to some time each month	22 (8.6)	268 (27.1)	34 (50.7)	803 (82.7)	**χ**^**2**^** = 125.49, * p* < .001** **Cramer’s* V* = .32**	**FET:** ***p* < .001**
Some time each week	113 (44.0)	562 (56.8)	25 (37.3)	158 (16.3)		
More or less daily	122 (47.5)	159 (16.1)	8 (11.9)	10 (1.0)		
*Time spent on pornography use/time*						
< 30 min	172 (66.9)	789 (79.8)	54 (80.6)	891 (91.8)	**χ**^**2**^** = 28.56, *p* < .001** **Cramer’s* V* = .15**	**FET:** ***p* = .002**
30**–**59 min	70 (27.2)	186 (18.8)	9 (13.4)	72 (7.4)		
≥ 1 h	15 (5.8)	14 (1.4)	4 (6.0)	8 (0.8)		
Ever used violent/coercive pornography	67 (26.1)	86 (8.7)	25 (37.3)	115 (11.8)	**χ**^**2**^** = 55.57, *p* < .001**	**χ**^**2**^** = 32.70, *p* < .001**
					***φ* = .21**	***φ* = .18**
Ever used sexual material involving children or animals	23 (8.9)	35 (3.5)	7 (10.4)	20 (2.1)	**χ**^**2**^** = 12.26, * p* < .001**	**FET: *p* = .001**
					***φ* = .10**	
Ever used sexual material involving children^a^	8 (3.1)	9 (0.9)	0 (0)	9 (0.9)		
Ever used sexual material involving animals^a^	22 (8.6)	33 (3.3)	7 (10.4)	15 (1.5)		

Statistics: Pearson’s chi-square statistics (χ2), Fisher’s Exact Test (FET), Phi (*φ*), Cramer’s *V*

^a^Only descriptives presented, due to low prevalence

Note. Bold values indicate statistically significant results (*p* < .05).

### Logistic Regression Analyses

Girls born outside Sweden were more than twice as likely to report PPU (cOR 2.05, 95% CI: [1.05–4.00], Table 5) compared to those born in Sweden. Additionally, the multivariate analysis indicated a potential difference in PPU among boys in theoretical versus vocational study programs (aOR 1.42, 95% CI [1.00–2.03], Table 5). The univariate and multivariable analyses of sexuality, along with health and abuse variables, aligned with group comparison results (Supplementary Material 1 and 2).

**Table 5:** Univariate and multivariable analyses of problematic pornography use and sociodemographic factors

	cOR [95% CI]		aOR [95% CI]^*^	
	Boys	Girls	Boys	Girls
*Living with both parents or alternating*				
Yes	0.94 [0.68**–**1.28]	1.05 [0.60**–**1.84]	1.00 [0.71**–**1.42]	1.24 [0.67**–**2.27]
No	Reference	Reference	Reference	Reference
Born in Sweden				
No	1.35 [0.87**–**2.09]	**2.05 [1.05–4.00]**	1.32 [0.83**–**2.10]	1.88 [0.92**–**3.84]
Yes	Reference	Reference	Reference	Reference
*Maternal employment*				
Unemployed	Reference	Reference	Reference	Reference
Employed	1.07 [0.69**–**1.66]	0.89 [0.44**–**1.83]	1.21 [0.76**–**1.92]	1.12 [0.52**–**2.42]
*Paternal employment*				
Unemployed	Reference	Reference	Reference	Reference
Employed	0.79 [0.52**–**1.20]	0.64 [0.35**–**1.20]	0.84 [0.93**–**1.34]	0.70 [0.34**–**1.44]
*Poor economic situation*				
No	Reference	Reference	Reference	Reference
Yes	1.60 [0.82**–**3.15]	1.73 [0.61**–**4.92]	1.58 [0.78**–**3.22]	1.38 [0.45**–**4.30]
*Study program*				
Theoretical	1.40 [0.98**–**1.99]	1.00 [0.57**–**1.76]	**1.42 [1.00–2.03]**	0.98 [0.55**–**1.73]
Individual	1.80 [0.85**–**3.79]	0.47 [0.06**–**3.62]	1.80 [0.85**–**3.81]	0.49 [0.06**–**3.78]
Vocational	Reference	Reference	Reference	Reference

^*^adjusted for all variables presented in the table

Note. Bold values indicate statistically significant results (*p* < .05).

Lastly, we explored which variables best predicted the probability of PPU (Table 6). For girls, the factors were being born outside Sweden (aOR 2.48, 95% CI [1.22–5.04]), having high-risk alcohol consumption (aOR 1.90, 95% CI [1.14–3.21]), and higher scores on TSCC (aOR 1.02, 95% CI [1.01–1.04]). For boys, less experience of vaginal sex (aOR 0.60, 95% CI [0.44–0.81]), more experience of sending sexually explicit images (aOR 1.71, 95% CI [1.23–2.36]), exposing someone else to nonconsensual sexual acts (aOR 3.69, 95% CI [1.54–8.85]), and higher scores on TSCC (aOR 1.02, 95% CI: [1.01–1.02]) predicted the likelihood of PPU.

**Table 6:** Final model derived from multivariable analyses of problematic pornography use

	aOR [95% CI]*	
	Boys	Girls
*Born in Sweden*		
No		**2.48 [1.22–5.04]**
Yes		Reference
*Study program*		
Theoretical	1.33 [0.92**–**1.92]	
Individual	1.66 [0.76**–**3.63]	
Vocational	Reference	
*Experience of vaginal sex*		
No	Reference	
Yes	**0.60 [0.44–0.81]**	
*Sexual debut *< *15 years*		
No		Reference
Yes		1.73 [0.95**–**3.15]
*Sending sexually explicit images*		
No	Reference	Reference
Yes	**1.71 [1.23–2.36]**	1.72 [0.98**–**3.04]
*Exposed someone else to nonconsensual sexual acts*		
No	Reference	
Yes	**3.69 [1.54–8.85]**	
*High-risk alcohol consumption*		
No		Reference
Yes		**1.90 [1.14–3.21]**
Difficulties with Emotion Regulation Scale (DERS-16)	1.01 [0.99**–**1.02]	1.00 [0.98**–**1.02]
Trauma Symptom Checklist for Children (TSCC)	**1.02 [1.01–1.02]**	**1.02 [1.01–1.04]**
*Multiple types of abuse*^a^		
No	Reference	Reference
Yes	1.05 [0.74**–**1.48]	0.92 [0.52**–**1.62]

^*^adjusted for all variables presented in the table

^a^Exposure to more than one type of abuse, including bullying

Note. Bold values indicate statistically significant results (*p* < .05).

## Discussion

In this study, we explored the prevalence and factors associated with PPU among a representative school-based sample of Swedish adolescents, grouped by gender. Our findings revealed a prevalence of PPU that aligns with previous research on youths (Jepsen & Brzank, 2022), with a higher occurrence in boys, but no association between PPU and socioeconomic status, contrasting with findings in Israeli adolescents (Efrati & Gola, 2018b).

Girls born outside Sweden demonstrated an increased likelihood of PPU, in line with research on CSBD and migration background (Briken et al., 2022). Girls with migrant experiences may face unique challenges, making them more susceptible to PPU. Considering the secular context of Swedish society, one explanation could be that girls raised in environments with stricter sexual norms and greater religiosity could experience a discrepancy between personal moral disapproval of pornography and actual pornography use, often referred to as moral incongruence. Moral incongruence is often related to religiosity (Grubbs et al., 2019) and may explain why 50% of girls with PPU reported only occasional pornography use, as elevated moral incongruence is linked to self-reported PPU, without objectively dysregulated use (Chen et al., 2022). Though our study did not directly assess this variable, it warrants further investigation.

Moral incongruence also affects boys, though potentially to a lesser extent, due to more permissible attitudes toward pornography (Petersen & Hyde, 2010). Overall, however, nearly half of boys with PPU watched pornography more or less daily, and individuals with PPU generally viewed it more frequently and for longer durations, consistent with findings that frequency is the strongest predictor of adult PPU (Bőthe et al., 2024a, 2024b).

Girls with PPU were more sexually experienced, with a higher number of partners and earlier initiation of sexual activity. Conversely, boys with PPU reported fewer sexual experiences; this aligns with findings that adolescents engaging exclusively in online sexual activities exhibit higher levels of CSB (Efrati & Amichai-Hamburger, 2021). Offline coercive sexual behaviors were also associated with PPU among boys, consistent with research on adults with CSB (Engel et al., 2019a, 2019b). Although this was only reported by 5% of boys with PPU, it was still 4 times more common compared to the reference group. Participants with PPU were more likely to have viewed violent and deviant content, which aligns with prior findings linking CSBD and high prevalence of paraphilias (Engel et al., 2019a, 2019b). Viewing sexual material involving children does not necessarily indicate sexual interest in children, given that participants were recently minors themselves and the circumstances of exposure are unclear. However, exposure to such material could influence normative attitudes. The use of violent material was more prevalent among girls than boys, contrasting with the findings of Engel et al. (2019a, 2019b). However, the scope for wider extrapolation is restricted by the limited number of participants. It may be that girls who engage with violent forms of pornography consequently report a heightened sense of loss of control regardless of overall usage levels. Nonetheless, the direction of causality between deviant content and PPU remains unclear; exposure to deviant material could contribute to perceived loss of control, or PPU individuals may seek such material due to their extensive and compulsive pornography use.

Although our study did not establish a direct correlation between PPU and formal psychiatric comorbidities, possibly due to the relatively young age of participants and limited time to establish formal diagnoses, we identified indications of mental health issues across both genders. This included difficulties with emotion regulation, self-harm, and for girls, higher rates of sex as self-injury, as well as drug use (reported by one-third of PPU girls) and alcohol use (reported by almost half of PPU girls). Individuals with PPU also demonstrated a higher prevalence of adverse childhood experiences, consistent with prior research on adults (Chatzittofis et al., 2017). While abusive experiences did not retain significance in the final model, they could be reflected in the elevated TSCC scores observed in the PPU groups.

Our primary focus was not a gender comparison; however, distinct gender profiles emerged. Among girls, the variables that best differentiated between PPU and non-PPU were: birth outside Sweden, high-risk alcohol consumption, and poor mental health as measured by the TSCC. Our findings suggest that girls with PPU exhibit more extensive risk behaviors, although their frequency of pornography use is not as high as that observed in boys. Self-injurious and risk-taking behaviors have been associated with the aftermath of sexual abuse, reflecting maladaptive coping strategies and possible reenactment processes (Van der Kolk, 1989; Zetterqvist et al., 2018). Among boys, less experience of vaginal sex, more sending of sexually explicit images, exposing others to nonconsensual sexual acts, and higher TSCC scores were the strongest predictors of PPU. These findings may suggest a profile of boys with social challenges and withdrawal; this aligns with the suggestion that adolescents with CSB are less social and more lonely (Efrati & Gola, 2018b). Despite some gender differences, the overall results suggest that youths with PPU exhibit coping deficits and may use pornography to regulate negative emotions, in line with findings in adults (Bőthe et al., 2024a, 2024b). Although studies of PPU in adolescents are limited (Testa et al., 2023), enhancing coping skills could benefit youths with PPU, as also recommended by Antons et al. (2023).

Several clinical implications emerge from these findings. The high prevalence of PPU in this sample should be taken into consideration by professionals in schools, youth clinics, and child and adolescent health services. Providing brief, destigmatizing information about PPU may help adolescents identify difficulties earlier and seek support. Clinical practitioners should be prepared to discuss pornography in a balanced manner and maintain a low threshold for initiating questions about such topics, especially when other risk behaviors or emotional difficulties are present. Assessments should also consider moral incongruence, general and sexual risk behaviors, and adverse childhood experiences. A particular challenge involves supporting young people who have both experienced adversity and are at higher risk of harming others.

### Limitations

This study has several limitations that should be considered when interpreting the results. Our measure of PPU, based on only two items previously used in earlier studies, may not fully capture the complexity of the construct. The survey was part of a broad epidemiological questionnaire covering multiple domains, which limited the scope for including more items. Future research could benefit from using more comprehensive scales to screen for PPU (Bőthe et al., 2021) or even CSBD, which may also manifest in other behaviors.

The cross-sectional design of the study limits our ability to draw conclusions about causality, directionality, and the stability of PPU over time. As noted in other studies, CSB and PPU are not stable constructs (Castro-Calvo et al., 2024; Štulhofer et al., 2020), highlighting the need for longitudinal studies. While the variables in the final model best predict PPU according to our data, they rely on self-reported data and are not necessarily the most meaningful or significant parameters in all contexts. There may be underlying psychological causes that we did not explore, such as personality traits, which could be important factors in PPU.

Although the COVID-19 pandemic affected adolescents’ mental health and increased periods of remote schooling (Svedin et al., 2021), Sweden did not implement strict lockdowns to the same extent as many other countries (Andersson & Jonung, 2024). While Swedish national data indicate that pornography use did not increase during this period (Zattoni et al., 2021), the possibility remains of some pandemic-related influences on our sample. Moreover, logistical difficulties during the pandemic required a pause in the data collection until a new digital solution was implemented, resulting in a lower response rate that may have affected our results.

Despite these limitations, this study contributes new evidence to a field in which adolescent PPU has been largely overlooked and has several notable strengths. The large, school-based sample enhances the generalizability of the findings and offers strong ecological validity, by capturing pornography use patterns in everyday adolescent life. Finally, the comprehensive assessment of sociodemographic, mental health, sexual behavior, and abuse variables allows for a nuanced examination of factors associated with PPU during a key developmental period. Further research should move beyond prevalence estimates by employing longitudinal designs and by investigating the gender-specific patterns indicated by our results, with replication needed across diverse cultural contexts. Future efforts should also focus on developing interventions for adolescents experiencing loss of control over their sexual behaviors.

### Conclusions

Our findings indicate that PPU is a significant concern, particularly among boys. However, clinicians should inquire about pornography use regardless of gender and avoid pathologizing sexual behaviors, as well as take a thorough history to identify vulnerabilities for PPU and enable early intervention. The link between PPU and emotional dysregulation, as well as trauma symptoms, also highlights the importance of addressing underlying psychological issues in prevention and treatment efforts.

## Supplementary Information

Below is the link to the electronic supplementary material.

Supplementary file1 (DOCX 18 KB)

## Data Availability

The ethical approvals for the data generated and analyzed in this manuscript do not allow for or include the opportunity to deposit the data in repositories. Code for data cleaning and analysis is available from the corresponding author on reasonable request.
