# Supplementary material for: Problematic Pornography Use Among Swedish Adolescents: Characteristics and Clinical Implications
Source: Arch Sex Behav. 2026 Oct 8;55(7):3149–61. doi: 10.1007/s10508-026-03544-9 (PMC13748086; doi:10.1007/s10508-026-03544-9)
Supplement: Supplementary file 1 [file 10508_2026_3544_MOESM1_ESM.docx]

**Supplement 1**

*Univariate and multivariable analyses of Problematic Pornography Use and sexuality*

|  | cOR [95% CI] | | aOR [95% CI]^*^ | |
| --- | --- | --- | --- | --- |
|  | Boys | Girls | Boys | Girls |
| Heterosexual identification  No  Yes | 1.40 [0.93**–**2.12]  Reference | 1.24 [0.70**–**2.20]  Reference | 1.02 [0.65**–**1.60]  Reference | 1.17 [0.65**–**2.11]  Reference |
| Number of sexual partners  0  1**–**5  ≥ 6 | 1.22 [0.92**–**1.63]  Reference  0.94 [0.58**–**1.52] | 0.99 [0.55**–**1.79]  Reference  **2.33 [1.20–4.52]** | 0.98 [0.42**–**2.28]  Reference  0.96 [0.56**–**1.64] | 0.75 [0.11**–**4.99]  Reference  1.50 [0.75**–**3.00] |
| Experience of oral sex  No  Yes | Reference  0.88 [0.67**–**1.16] | Reference  1.46 [0.86**–**2.48] | Reference  1.09 [0.58**–**2.03] | Reference  1.08 [0.24**–**4.75] |
| Experience of vaginal sex  No  Yes | Reference  **0.72 [0.55–0.95]** | Reference  1.17 [0.69**–**1.98] | Reference  **0.53 [0.28–1.00]** | Reference  0.36 [0.10**–**1.26] |
| Experience of anal sex  No  Yes | Reference  1.16 [0.82**–**1.65] | Reference  1.56 [0.90**–**2.69] | Reference  1.15 [0.76**–**1.76] | Reference  0.90 [0.47**–**1.72] |
| Sexual debut < 15 years  No  Yes | Reference  0.80 [0.50**–**1.27] | Reference  **2.86 [1.67–4.93]** | Reference  0.76 [0.44**–**1.32] | Reference  **2.17 [1.11–4.24]** |
| Sending sexually explicit images  No  Yes | Reference  **1.84 [1.38–2.44]** | Reference  **2.57 [1.54–4.29]** | Reference  **2.13 [1.54–2.95]** | Reference  **2.71 [1.43–5.15]** |
| History of chlamydia  No  Yes | Reference  1.45 [0.39**–**5.38] | Reference  2.52 [0.88**–**7.26] | Reference  2.00 [0.48**–**8.42] | Reference  1.36 [0.44**–**4.25] |
| Abortion (self or partner)  No  Yes | Reference  0.44 [0.13**–**1.46] | Reference  2.48 [0.74**–**8.32] | Reference  0.37 [0.10**–**1.34] | Reference  1.40 [0.39**–**5.05] |
| Exposed someone else to nonconsensual sexual acts  No  Yes | Reference  **4.88 [2.20–10.83]** | Reference  1.32 [0.17**–**9.98] | Reference  **4.38 [1.90–10.08]** | Reference  1.05 [0.14**–**8.26] |
| * adjusted for all variables presented in the table | | | | |

**Supplement 2**

*Univariate and multivariable analyses of Problematic Pornography Use and* *health and abusive experiences*

|  | cOR [95% CI] | | aOR [95% CI]^*^ | |
| --- | --- | --- | --- | --- |
|  | Boys | Girls | Boys | Girls |
| **Health** |  |  |  |  |
| High-risk alcohol consumption  No  Yes | Reference  0.94 [0.70**–**1.26] | Reference  **2.26 [1.38–3.70]** | Reference  1.00 [0.73**–**1.38] | Reference  **1.84 [1.08–3.14]** |
| Lifetime drug use  No  Yes | Reference  1.00 [0.74**–**1.36] | Reference  **2.58 [1.54–4.32]** | Reference  0.80 [0.57**–**1.13] | Reference  1.46 [0.80**–**2.66] |
| Psychiatric/neuropsychiatric comorbidity  No  Yes | Reference  1.37 [0.94**–**1.99] | Reference  1.41 [0.81**–**2.45] | Reference  0.76 [0.49**–**1.19] | Reference  0.75 [0.39**–**1.41] |
| Nonsuicidal self-injury  Yes  No | **1.61 [1.14–2.26]**  Reference | **1.98 [1.21–3.23]**  Reference | 0.94 [0.62**–**1.41]  Reference | 0.88 [0.47**–**1.64]  Reference |
| Use of sex as self-injury  Yes  No | 1.18 [0.48**–**2.95]  Reference | **3.57 [1.98–6.43]**  Reference | 0.47 [0.17**–**1.33]  Reference | 1.75 [0.87**–**3.54]  Reference |
| Difficulties with Emotion Regulation Scale  (DERS-16) | **1.03 [1.02–1.04]** | **1.03 [1.01–1.04]** | **1.01 [1.00–1.02]** | 1.00 [0.98**–**1.02] |
| Trauma Symptom Checklist for Children  (TSCC) | **1.02 [1.02–1.03]** | **1.03 [1.02–1.04]** | **1.02 [1.01–1.03]** | **1.02 [1.01–1.04]** |
| **Abusive experiences^a^** | | | | |
| Exposure to bullying  No  Yes | Reference  **1.73 [1.31–2.29]** | Reference  **2.18 [1.22–3.91]** | Reference  1.20 [0.86**–**1.68] | Reference  1.75 [0.87**–**3.56] |
| Emotional abuse  No  Yes | Reference  **1.61 [1.14–2.26]** | Reference  1.66 [0.96**–**2.87] | Reference  0.92 [0.55**–**1.53] | Reference  0.74 [0.35**–**1.59] |
| Physical abuse  No  Yes | Reference  **1.67 [1.21–2.31]** | Reference  **1.98 [1.15–3.42]** | Reference  1.09 [0.66**–**1.79] | Reference  1.32 [0.63**–**2.74] |
| Exposed to any form of sexual abuse  No  Yes | Reference  1.34 [0.89**–**2.01] | Reference  **2.03 [1.25–3.32]** | Reference  1.10 [0.67**–**1.81] | Reference  1.19 [0.53**–**2.68] |
| Multiple types of abuse^b^  No  Yes | Reference  **1.77 [1.31–2.38]** | Reference  **1.98 [1.21–3.23]** | Reference  1.13 [0.59**–**2.16] | Reference  0.75 [0.27**–**2.04] |
| Grooming  No  Yes | Reference  1.37 [0.90**–**2.08] | Reference  **1.73 [1.06–2.82]** | Reference  0.90 [0.56**–**1.45] | Reference  1.06 [0.61**–**1.82] |
| * adjusted for all variables presented in the table  ^a^ Exposure  ^b^ Exposure to more than one type of abuse above, including bullying | | | | |
